# When quick response codes didn’t do the trick

**DOI:** 10.1007/s40037-020-00572-6

**Published:** 2020-04-06

**Authors:** Gabrielle Walcott-Bedeau, Kazzara Raeburn, Dirk Burkhardt, Mark Clunes

**Affiliations:** 1 Department of Physiology, Neuroscience and Behavioural Sciences, St George’s University, St George’s, Grenada; 2 Department of Anatomical Sciences, St George’s University, St George’s, Grenada

**Keywords:** QR codes, Self-directed learning, Smartphone, Learning objectives

## Abstract

Medical education programs in the United States or Canada comply with the Liaison Committee on medical education standards to ensure their graduates provide proficient medical care. One standard includes student development as a lifelong learner. The competency of lifelong learning is developed through self-directed activities such as students evaluating their learning objectives and resources without external help.

Quick response (QR) codes were the technological tools introduced in a traditional medical institution to enhance students’ self-directed initiative to tap resources. Relevant lecture objectives and other information such as supplemental discipline content, reading assignments and web-based link resources were embedded into codes and ‘pasted’ onto all pages of their course PDF handouts. It was anticipated that most students had access to smart phones to conveniently scan the codes and retrieve the information.

However, an in-class survey conducted showed that only 30% of the students found the QR codes useful. Further questioning revealed that some students just didn’t know how to use the codes or didn’t think the information embedded was worth the effort to decrypt. Although students were tech-savvy in the social and entertainment realms, they were not adept in the use of technology for educational purposes.

QR codes presented several theoretical, pedagogical advantages to enhance experiential and self-directed learning. However, implementation among students, in a traditional classroom, required prior instructions on usage. Student feedback was also imperative when introducing novel, innovative tools like QR codes.

## The story

A medical education program achieves and maintains accreditation by ensuring that their graduates have developed skills to become lifelong learners and to provide proficient medical care. The Liaison Committee on Medical Education (LCME) regularly reviews members of the medical education community (United States or Canada) for compliance with the standards that govern the function and structure of medical schools. One of these standards is that the medical curriculum includes self-directed learning experiences [1]. Our institution was preparing for re-accreditation and wanted to ensure compliance with all the LCME standards. A team comprising both senior and junior members of faculty gathered for a brainstorming session. The members of the team were tasked with executing a project that increased the opportunities for our medical students to develop the skills of lifelong learning. In fact, self-directed learning is a skill required if a medical student is to become a lifelong learner [2].

Lifelong learners must be able to evaluate their resources and the learning objectives of the task without any external help [3]. At our institution, each teaching faculty includes an introductory power-point slide that lists the learning objectives of the discipline content presented during that lecture. PDF copies of these power-point slides are published in the student resource folder using an online interface. During the brainstorming session, the team suggested that faculty can facilitate student development of this competency by mapping the learning objectives closer to the relevant content on their power-point lecture slides.

Traditionally, the published PDF handout versions of the faculty’s power-point slides do not only contain lecture objectives but also information such as supplemental discipline content, reading assignments, and web-based link resources. However, the amount of information that a lecturer can include on a single power-point slide is limited by the available ‘white space’ on that slide. For example, if the faculty’s lecture slide is detailing the physiological changes associated with cardiovascular murmurs, they may also wish to include the cardiac cycle associations, images of the pressure-volume loop changes, and even a URL link to the website to hear these abnormal sounds. Inclusion of all that information on a single slide can make it appear crowded and leave students feeling overwhelmed. As such, educators must find an innovative approach to their delivery of large volumes of information to the students.

The Quick Response (QR) code is a two-dimensional bar code. It can be generated by many ‘easy to use’ tools on the internet. A quick google search generates many websites that have made available free QR code generators. Information such as long strings of multi-lingual text, images, hyperlinks to any media can be embedded into the matrix design. In addition to word text, the major employ of QR codes is ease of direct activation of the URL links. The black and white QR code image is saved and can be affixed to any print media. The information can be quickly retrieved when a mobile phone or smart device equipped with a camera and QR code reader scans the symbol. The surge in recent years of the camera equipped/QR code reader smartphones/devices and their widespread use makes the QR code an accessible method of encoding information[4]. Albeit, in situations when students did not have QR code equipped devices, a QR code reader app can be downloaded onto their devices to facilitate in-class cryptography. The ease of creation and retrieval of information makes QR codes an ideal educational tool for the innovative medical educator [4].

The faculty team suggested that any activity that encouraged students to use their initiative and seek out resources will promote self-directed learning. The proposal further specified that lecture objectives and other supplemental information can be ciphered into the QR code symbol and presented to students on a small area on each slide of the published lecture material. Faculty members were excited about the utility of the smartphones in the classroom. Students could scan the QR codes from their PDF slides displayed on their laptop screens. The brainstorming team anticipated that the QR codes would enhance the students’ self-directed learning experience.

## The activity

Year One medical students in a medical physiology course, (*n* = 482; Fall 2017) were given PDF lecture handouts with QR codes on each slide. Prior to the commencement of the semester, the teaching faculty were briefed on how to create the QR codes using any of the free QR code generator software available online. Most faculty preferred to use https://www.the-qrcode-generator.com [5]. The learning objectives and other information specific to the course such as the associated readings from the reference text, research articles or additional web resources could have been embedded into the code. When the code was scanned a single page document with bulleted points containing word text was opened. The QR code was created and pasted in a free white space on the power-point slide, generally alongside the lecture title. This lecture slide is saved as a PDF version with the embedded QR code for students to scan. The modified lecture slides were released with the newly embedded QR codes at the start of the new term (Fall 2017). Students were not given instructions on how to use the QR codes. Neither were they informed that the QR codes were on the slides to embed information like their lecture objectives. It was assumed that when our students accessed these lecture slides via PDF files delivered by the course management system, their curiosity about the ‘black and white square’ image would direct them to the additional information. It was believed that the students would automatically know what to do to decode the image.

After the release of the new slides, around mid-semester, the brainstorming team was curious if the QR codes were used or were helpful. In order to collect some data, a single multiple-choice question survey was conducted in a random interactive session. The faculty member collected the data using the Turning Point software interface. Students used an interactive clicker device that generated polling responses to the survey question.

## Surprising outcomes

A total of 482 active participants in the interactive multiple-choice question session where asked to agree or disagree with the statement “I found the QR code useful”. The students were not given any definition of the term useful. Of the total number of participants, less than 30% responded that they found the QR codes useful. Alarmingly, 68% of the students disagreed with the usefulness of the QR codes (See Tab. 1). The negative feedback suggested to the team that a smaller sample of students should be asked further questions informally. Given the theoretical advantages and novelty of the QR codes, why didn’t the students find the codes useful?

**Table 1:** Student responses to an in-class clicker question on the usage of the Quick Response codes

I have found the QR codes useful	Number of students	Responses (%)
Strongly agree	41	9
Agree	93	19
Strongly disagree	164	34
Disagree	184	38
*Totals*	*482*	*100*

## Lessons learned

The investigators wanted to illicit an explanation why the QR codes were not utilized. Follow-up informal discussions with a few students from the class were conducted. Firstly, some students did not find the QR codes useful because “They did not know how to ‘read’ the codes”. Despite being in existence for decades, scanning QR codes was not ‘second nature’ for the students. Literature suggests that although students have technological experience in social and entertainment realms, they are not ‘natives’ in the use of technology in an educational manner [6].

**Fig. 1:**
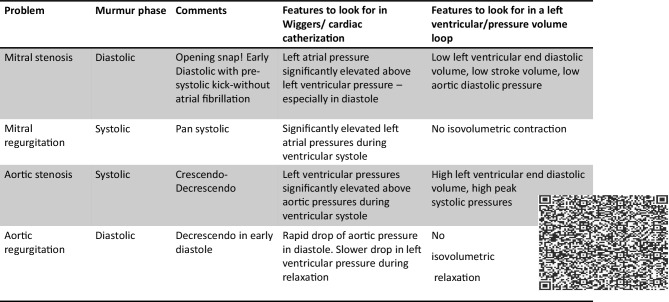
A sample lecture slide from the cardiovascular module in the physiology course. The Quick Response code has embedded the learning objective, the reference textbook with page numbers and supplemental information websites. The QR codes can be placed anywhere in the slide and the size reduced as necessary. However, all QR codes should be tested on the published lecture slide material to ensure the code is easily scanned [7]

Secondly, the students revealed, “They did not know what the QR codes were about”. Students were unaware that each lecture slide was mapped to a learning objective and that some QR codes contained additional information related to their lecture content (See Fig. 1). Even when curiosity superseded, it did not seem worth the effort to scan the codes on every single lecture slide to find at times *only* lecture objectives.

Nevertheless, some students were able to scan the codes. There were a few technical errors in interpreting the codes. It seems that when the image was resized in some instances to ‘fit’ unto the slide, it distorted the code. Additionally, scans would only work on codes that were a specific resolution and distance away from the smart device’s camera. Students had to tilt their devices in many angles to retrieve the linked information.

Despite, negative response from students, the brainstorming team predicted that student interaction with the QR codes would have presented several theoretical advantages. These included:

- Opportunity for self-directed learning, with reading assignments mapped directly unto the page with the relevant content;
- Ubiquity of smartphones translates to the immediate access of students to additional encoded information;
- If students did not have access to a camera-enabled, many QR code reader apps are available for free;
- Large quantities of supplemental information can be encoded within the QR code;
- Information retrieval via scanning a QR code is easier than typing the web addresses;
- Faculty can map their curriculum and lecture content by embedding learning objectives into the QR codes;
- The design of the QR code pattern adds some visual appeal to the document.

Two hasty generalizations were made. The first was that in an age of multi-taskers and technologically advanced classrooms, students would not need an instructional guide on the utilization of ‘simple’ QR codes. The second was that students would appreciate being given lecture objectives and additional information in a technologically novel way.

Although technological tools in the classroom would promote engagement of the learners, implementation would require the learners to be aware of the tool, which requires provision of some sort of instruction by the teacher.

## Moral of the story

A novel tech-savvy idea like QR codes should have encouraged ‘all’ students to conveniently scan the codes and be recipients of the additional information prepared by the lecturer. This was not the case!

While QR codes are a technological tool used to enhance active thinking and self-directed learning, it may not create the change in aspects of learning, productivity and efficiency [6]. If the use of such novel ideas is to be implemented in the traditional classroom, it should be implemented as a pilot. Additionally, our findings indicate that novel instructional items, such as QR codes, must be accompanied by instruction, as well as allowances for feedback. In addition to QR codes, other digital tools are virtual learning, gamification, cloud computing, digital library and analytics. These pedagogical tools enhance experiential and self-directed learning which can lead to a positive active-learning environment in a traditional classroom. Once the medical student inculcated these habits that aid self-directed experiences, they will foster life-long learning.
